# Below-ground interspecific competition for water in a rubber agroforestry system may enhance water utilization in plants

**DOI:** 10.1038/srep19502

**Published:** 2016-01-19

**Authors:** Junen Wu, Wenjie Liu, Chunfeng Chen

**Affiliations:** 1 Key Laboratory of Tropical Forest Ecology, Xishuangbanna Tropical Botanical Garden, Chinese Academy of Sciences, Menglun, Yunnan, 666303, China; 2 University of Chinese Academy of Sciences, Beijing 100049, China

## Abstract

Rubber-based (*Hevea brasiliensis*) agroforestry systems are regarded as the best way to improve the sustainability of rubber monocultures, but few reports have examined water use in such systems. Accordingly, we tested whether interplanting facilitates water utilization of rubber trees using stable isotope (δD, δ^18^O, and δ^13^C) methods and by measuring soil water content (SWC), shoot potential, and leaf C and N concentrations in a *Hevea-Flemingia* agroforestry system in Xishuangbanna, southwestern China. We detected a big difference in the utilization of different soil layer water between both species in this agroforestry system, as evidenced by the opposite seasonal fluctuations in both δD and δ^18^O in stem water. However, similar predawn shoot potential of rubber trees at both sites demonstrating that the interplanted species did not affect the water requirements of rubber trees greatly. Rubber trees with higher δ^13^C and more stable physiological indexes in this agroforestry system showed higher water use efficiency (WUE) and tolerance ability, and the SWC results suggested this agroforestry is conductive to water conservation. Our results clearly indicated that intercropping legume plants with rubber trees can benefit rubber trees own higher N supply, increase their WUE and better utilize soil water of each soil layer.

The effects of humans are at least as important as natural forces in shaping geological, ecological, and environmental patterns. The pervasiveness, magnitude, and variety of human impacts leave little doubt that we are currently in the Anthropocene, and these impacts are particularly prominent in developing countries^1^.

The rubber tree (*Hevea brasiliensis*) is a perennial crop that is native to the tropical rainforest of the Amazon Basin; it has both economic and social importance in many developing countries^2^ (particularly in tropical and subtropical areas). The ideal habitat for the rubber tree is characterized by low variation in air temperature (24–28 °C) and precipitation (monthly rainfall greater than 100 mm) throughout the year in areas of low latitude and elevation^3^,^4^,^5^. The demand for both natural and synthetic rubber is increasing owing to the development of tire manufacturing (accounting for 70% of rubber consumption) and approximately 44% of worldwide rubber is produced naturally from *H. brasiliensis*; accordingly, natural rubber is a key product in tropical regions. Therefore, people, especially smallholders who contribute 80% of the global latex production^6^, have extended rubber plantations to higher latitudes and altitudes. The majority of natural forests have been converted to monoculture rubber plantations^7^,^8^.

The loss of primary and secondary natural forests is particularly serious in the major rubber production areas of Southeast Asia. In the Yunnan province of China, rubber monoculture (Rm) covered more than 400,000 ha in 2009, or 20% of the land in Xishuangbanna, since the introduction of rubber in the 1950 s^8^. Compared to primary tropical forests, rubber monoculture is associated with significantly lower biodiversity^9^,^10^ and total biomass carbon stock^11^, negative hydrological consequences^12^,^13^,^14^, and increased pests. Present planting patterns also influence the latex yield, growth, and health of rubber trees, especially in the dry season^5^. Additionally, environment is polluted by pesticides, chemical fertilizers, herbicides, and other chemical drugs. These negative impacts have worsened as the number of extreme weather and climate events has increased (e.g., the severe drought in Yunnan province in 2010). The rubber agroforestry system, which combines agricultural and forestry technologies to create a more diverse, productive, healthy, and sustainable land-use system, is a promising and practical way to minimize such issues^15^. Not only that, rubber agroforestry system also can facilitate the diversification of agricultural products, promote faster returns on investment, and reduce the breakeven point since fluctuations in rubber prices have been a serious problem for growers^16^.

However, associations between the traits of mature rubber trees that are grown as the main crop and those of other crops are poorly documented, especially with respect to water use^11^. Rubber trees have been referred to as water pumps because they are associated with water depletion at the basin scale^14^. Rubber plantations can cause excess surface run-off during the rainy season^12^, leading to land degradation by erosion, waterlogging, and salinization^17^. Even worse, several decades of rubber tree planting in Xishuangbanna has resulted in reduced streamflow and dried up wells in many villages. Dry season water shortages, which seldom occurred in the past, even during the driest years, are now frequently experienced by local populations^8^,^12^. As the most important component of the rubber agroforestry system, the distribution and accessibility of water greatly affects plant growth and survival^18^. Currently, tracing sources of water utilized by rubber trees in rubber agroforestry systems is urgently needed^11^,^19^.

As a traditional Chinese medicine with various therapeutic purposes^20^, *Flemingia macrophylla* is widely planted in Xishuangbanna. It is a leguminous, perennial, leafy shrub and is widely used in agriculture, for crop improvement, and as fodder. Due to its low rate of leaf decomposition, dense growth, moderate drought tolerance, ability to withstand occasional flooding, and coppicing ability, it is commonly used for erosion and weed control, nitrogen fixing, moisture conservation, reduction of soil temperature, and as a windbreak^21^. In order to realize the beneficial effects of *F. macrophylla* on rubber plantations with respect to water use, we investigated the interspecific and intraspecific differences and variation in *Hevea-Flemingia* agroforestry systems (HFAs) among seasons. We measured the stable isotope ratios (δD and δ^18^O) of water in soil, rain, and plant tissue samples to quantitatively distinguish plant water sources. We also measured leaf δ^13^C and the soil water content (SWC) over the course of a rainy/dry season cycle (2013–2014) to compare the interspecific and intraspecific water use efficiency (WUE) and moisture conservation ability of the system, respectively. In addition, we measured the leaf C and N concentrations and shoot water potential to characterize the ecophysiological properties of the plants. We hypothesized that (i) rubber agroforestry systems maintain much more soil water than rubber monoculture systems; (ii) interplanting could improve the WUE and productivity of rubber trees via species interactions; and (iii) the rubber plants and the interplanted species may extract water from different sources.

## Results

### Precipitation and air temperature

**Figure 1:**
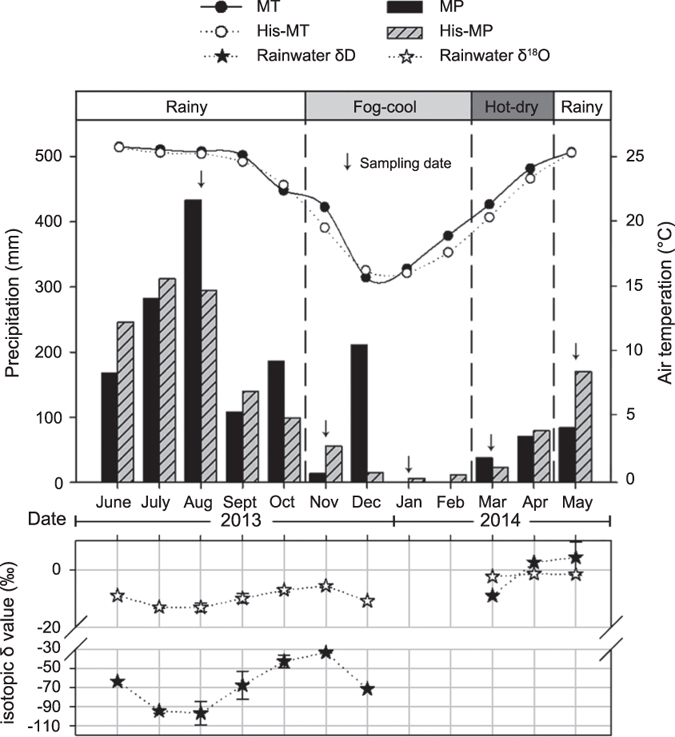
Monthly precipitation distribution and mean air temperature during the investigation period (historical data from XTBG ecological station). MT indicates monthly mean air temperature, and His-MT indicates historical monthly mean air temperature from 1969 to 2014; MP indicates monthly precipitation, and His-MP indicates historical monthly precipitation from 1969 to 2014. The stippled bars at the top of the panel indicate the season. Vertical arrows indicate the sampling date.

The total precipitation during the investigation period (June 2013–May 2014) was 1,594.5 mm, which was higher than the long-term mean (1,454.3 mm), but the seasonal variation was high (Fig. 1). The monthly mean air temperature was significantly higher than the long-term mean (*t* = 2.340, *P* = 0.039). It gradually dropped before January 2014, and then rose. An extreme weather event characterized by a sustained low temperature and strong storms, resulting in 211 mm of monthly precipitation, occurred in December 2013 and was followed by a dry spell of more than 2 months without any rainfall. Rainwater δD and δ^18^O varied among seasons (δD ranged from −107.42‰ to 2.25‰, and δ^18^O ranged from −14.59‰ to −1.26‰), and was lower in the rainy season than the dry season (*P* < 0.001). The observed variation was mainly attributed to the type of rainfall event; as expected, water collected during relatively heavy rainfall events had lower values, while lighter rainfall events showed more enrichment (i.e., higher values)^22^. In our study, we found a significant correlation between precipitation and rain water isotope values (*P* < 0.001), i.e., the correlation coefficients were −0.858 for δD and −0.853 for δ^18^O (Fig. 1).

### Soil water content and isotopic compositions of soil water and plant xylem water

**Figure 2:**
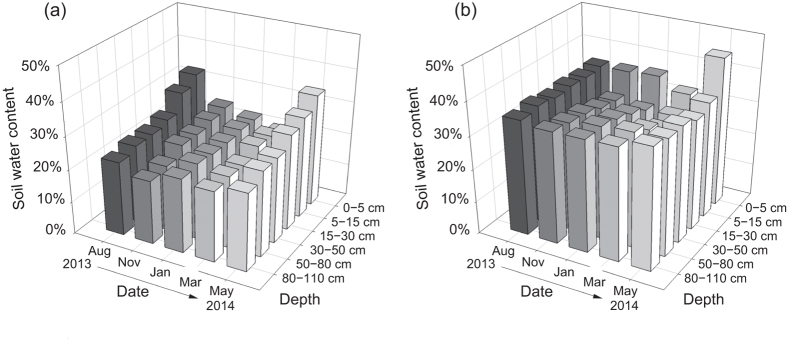
Soil water content (SWC) of each soil layer and seasonal variation for (**a**) rubber monoculture (Rm) and (**b**) the *Hevea-Flemingia* agroforestry system (HFAs).

SWC at both sites exhibited pronounced seasonal variation (*F* = 36.304, *P* < 0.01), with lower values observed in the dry season and higher values in the rainy season (Fig. 2). However, SWC in samples from below 30 cm at both sites did not show significant seasonal variation. In HFAs, the SWC of soil layers above 5 cm in depth was always significantly higher than that of other layers, and soil layers ranging from 5 cm to 30 cm showed a significantly lower SWC relative to other layers (*P* < 0.01). In addition, SWC in the layers below 30 cm in depth at both sites showed minimal seasonal variation from November 2013 to March 2014. SWC in HFAs was always significantly higher than in Rm during the investigating period (*F* = 662.127, *P* < 0.01).

**Figure 3:**
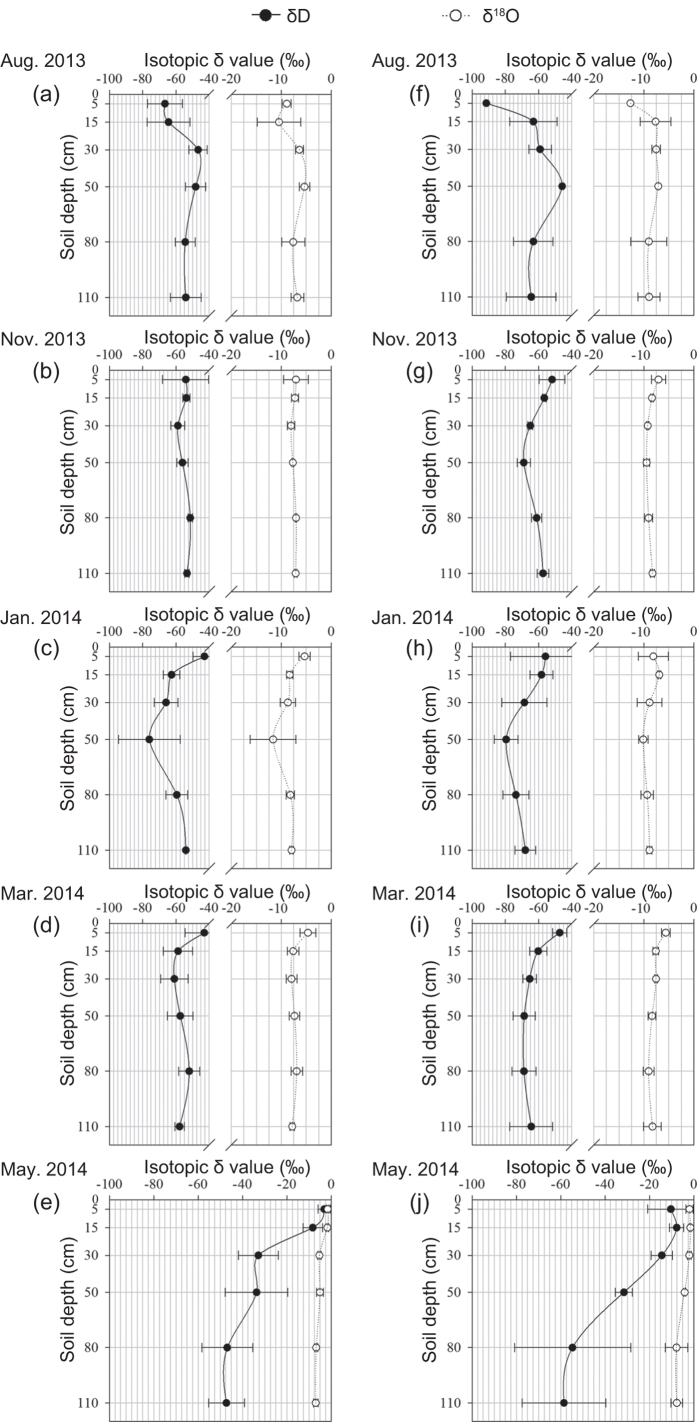
Isotopic composition of water and its gradient variation within soil profiles of (**a–e**) rubber monoculture and (**f–j**) the *Hevea-Flemingia* agroforestry system. Data are expressed as mean ± s.d.

Soil water δD and δ^18^O values showed significant differences among seasons, sites, and depths (*P* < 0.01). However, at depths of greater than 15 cm in Rm and greater than 30 cm in HFAs, minimal differences were observed. In addition, seasonal variation in the soil water isotope composition was not significant from August 2013 to January 2014 in Rm or from August 2013 to March 2014 in HFAs (Fig. 3). In contrast, soil water δD and δ^18^ O values were higher in Rm sites than HFAs (*P* < 0.01).

**Figure 4:**
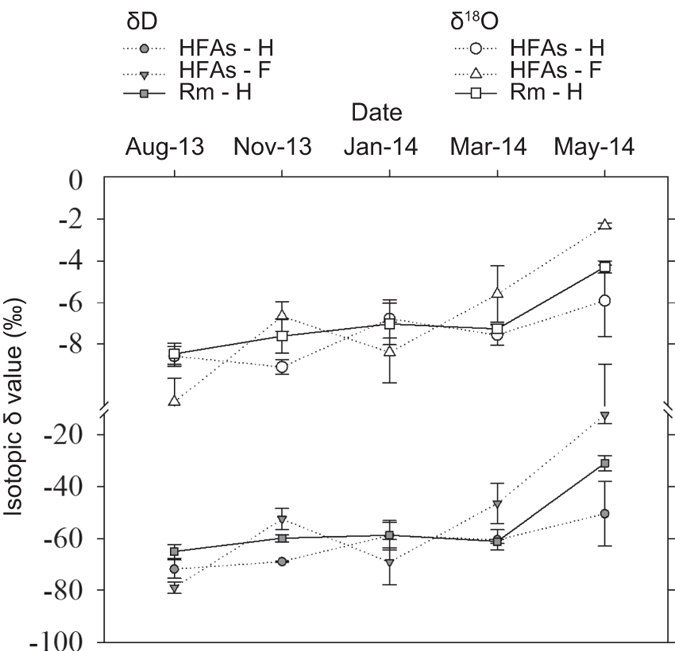
Seasonal variation in δD and δ^18^O values for plant stem (xylem) water. Rm-H indicates *Hevea brasiliensis* in Rm, HFAs-H indicates *H. brasiliensis* in HFAs, and HFAs-F indicates *F. macrophylla* in HFAs. Data are expressed as mean ± s.d.

For plant xylem water of rubber trees, δD and δ^18^O differed significantly among sites and seasons (*P* < 0.01). In HFAs, xylem water δD and δ^18^O exhibited the opposite trend with respect to seasonal fluctuations for both plant species (Fig. 4). In addition, the isotopic compositions of plant xylem water did not differ significantly from November 2013 to January 2014 in Rm, and the same was true of soil water isotope compositions. Therefore, we concluded that rubber trees in Rm sites utilized the same water source during this period, and the IsoSource model verified this inference (Fig. 6).

**Figure 5:**
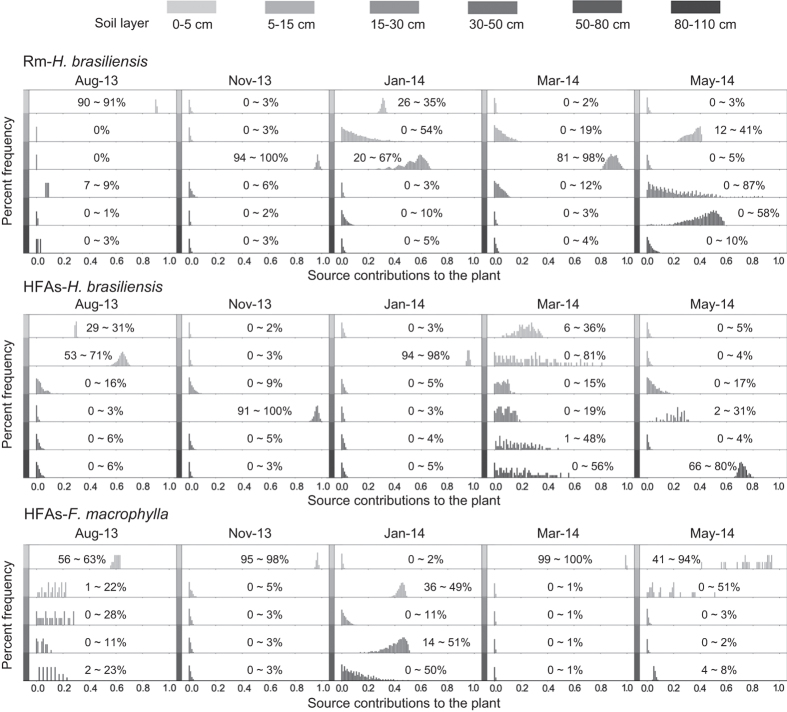
Relative water use for various sources for rubber trees and interplanted species at both sites during the investigation. The contributions were calculated for all model iterations (in 1% increments), and are expressed as percent frequencies of all possible solutions. The range of potential water source contributions is labeled. The stippled bars at the top of the panel indicate soil layers of different depths. Rm indicates rubber monoculture, HFAs indicates *Hevea-Flemingia* agroforestry system.

**Figure 6:**
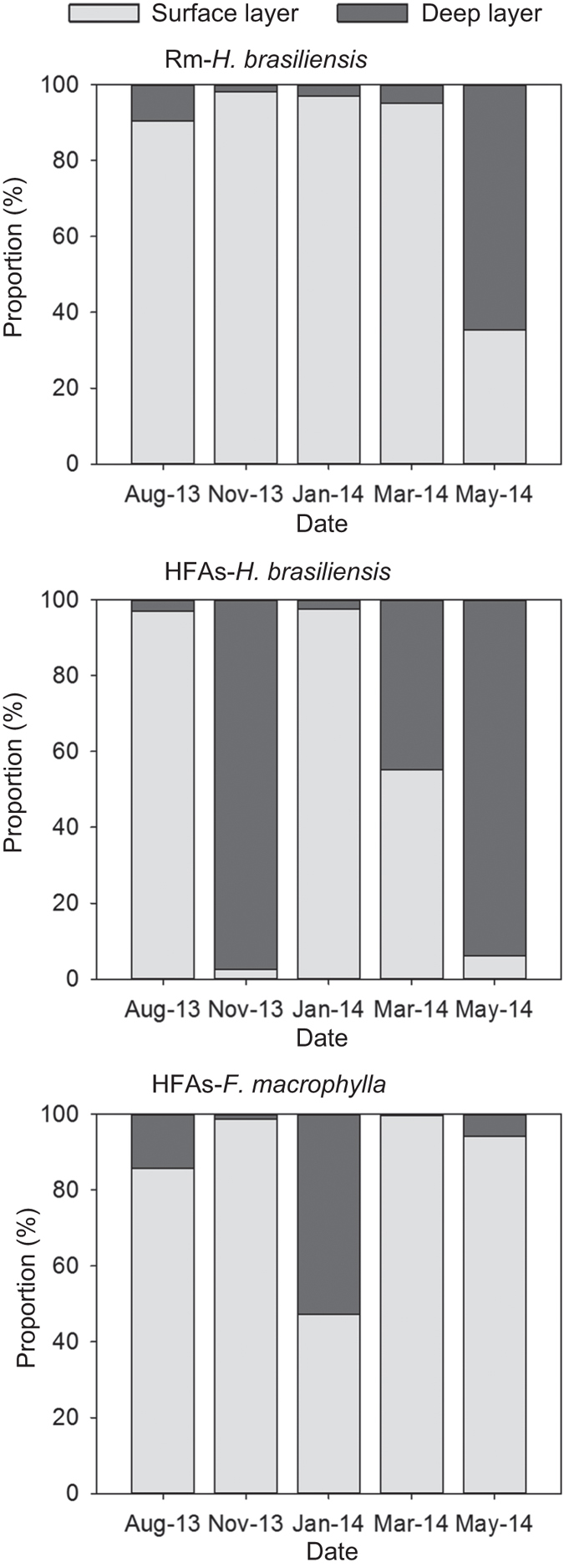
Mean contribution of various water sources to the xylem sap of rubber trees and interplanted species. The surface layer contained soil layers above 30 cm in depth, and the deep layer contained soil at 30−110 cm depth. See Fig. 5 for abbreviations

Finally, we used an IsoSource model^23^ to calculate the relative contributions of each water source (Fig. 5) and simplified the mixing analyses using an a posteriori method to combine sources owing to the overabundance of sources (i.e., 6 sources)^24^. Specifically, we divided the soil layer into two parts; layers above 30 cm in depth were combined as the surface layer and others were combined as the deep layer. This division was selected because the soil water isotope values in deep layers did not differ in multiple comparison analyses. Finally, we quantitatively defined the contribution of each water source to plants (Fig. 6). Rubber trees in Rm sites relied heavily on surface soil water (90.3–98.1%) before May 2014, but absorbed deep soil water (64.9%) in May 2014 owing to soil drought stress. Rubber trees in HFAs exhibited flexible switching of water sources because *F. macrophylla* relied mainly on surface soil water (47.3–99.6%).

### Plant leaf δ^13^C, carbon (C) and nutrient (N) concentrations, and shoot water potential

**Figure 7:**
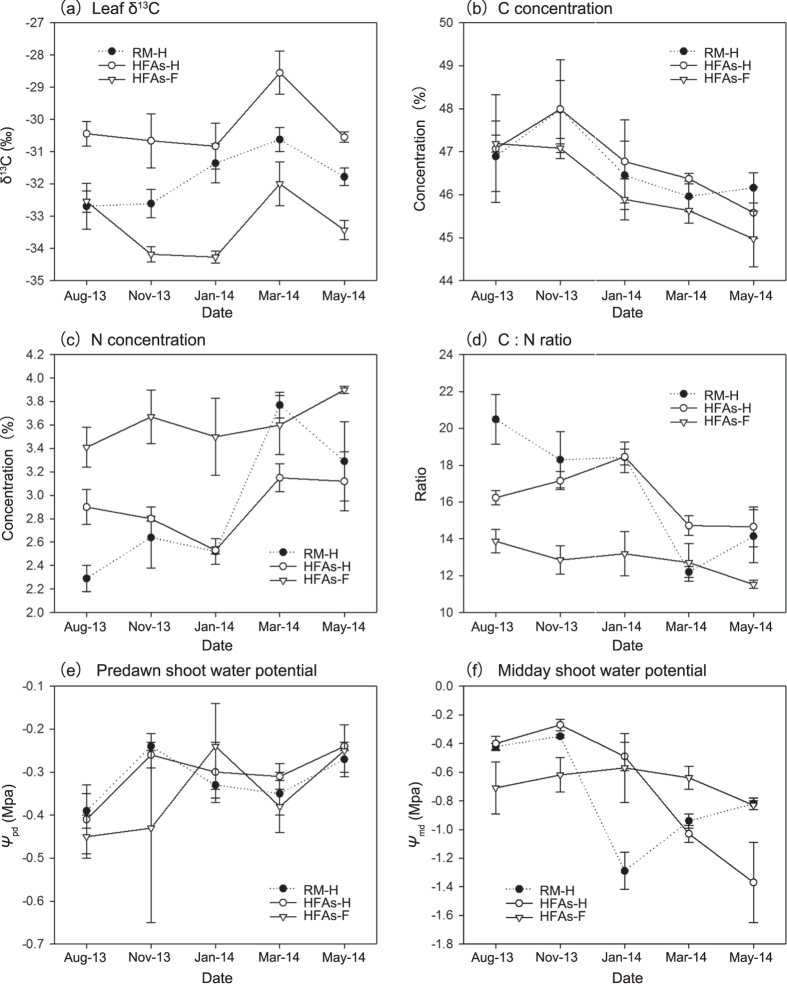
Leaf δ^13^C (**a**), C and N concentrations (**b,c**), C:N ratio (**d**), and shoot water potential (**e,f**) during the investigation period. See Fig. 4 for abbreviations. Data are expressed as mean ± s.d.

The mean leaf δ^13^C values of rubber trees were −31.81‰ and −30.21‰ in Rm and HFAs sites, respectively, and −33.28‰ for *F. macrophylla* in HFAs. These results indicated that all plants in our study belonged to C_3_ photosynthesis plant since their leaf δ^13^C ranged from −20 to −34‰^25^. Accordingly, the δ^13^C value was a reliable index for comparisons of WUE among plants. Leaf δ^13^C of rubber trees was significantly higher in HFAs than Rm for the duration of the experiment (*P* < 0.01), and the same was true of the interplanted species (Fig. 7a).

All rubber trees properties at both sites (except the C concentration of rubber trees in HFAs) showed significant seasonal variation (*P* < 0.01); however, the differences in rubber tree parameters among sites (except δ^13^C) were not obvious (Table 1). The interaction between season and site for the N concentration, C:N ratio, and *Ψ*_md_ for rubber trees was significant (*P* < 0.01, see Table 1A) and a simple effects analysis showed that these observed values changed more extensively among seasons for rubber trees in Rm than those in HFAs (Fig. 7c,d,f). Specifically, the N concentration of Rm rubber trees was significantly lower before January 2014 and significantly higher in March 2014 relative to that of HFAs rubber trees (Fig. 7c,d), and the opposite pattern was observed for the C:N ratio. In addition, *Ψ*_md_ of rubber trees was significantly higher in HFAs than Rm from January 2014 to March 2014 (*P* < 0.01). The *Ψ*_pd_ of HFAs rubber trees showed slight seasonal oscillations (Fig. 7e), but neither *Ψ*_pd_ nor *Ψ*_md_ of *F. macrophylla* in HFAs showed seasonal variation during the investigation (*P* > 0.05, see Table 1E).

## Discussion

The sampling dates were clearly representative of each season. However, the continuous, intense rain that occurred in November 2013 might disturb this climate pattern. Changes in the regional rainfall pattern are predicted to greatly affect the water balance of the ecosystem^18^, resulting in changes in the ecophysiological functions of plants. Notably, a decreased intensity and prolonged duration of drought were observed in the dry season.

In the fog-cool season, a high frequency of heavy-radiation fog always appears at night and in the morning; tree leaves are covered with fog drops and the soil is continuously wet. A previous study in this region suggested that fog-drip water is important for shallow soil^26^, and contains water that was evaporated and recycled from the river and soil, and water from forest evapotranspiration^12^. Therefore, this rain event might transport a large amount of water to the local water vapor cycle system, and the soil could maintain higher moisture than usual in the dry season when the systems are in equilibrium. In addition, rubber trees exhibit defoliation, which is the annual shedding of senescent leaves that renders trees wholly or partially leafless for about 1–2 weeks in January or February^27^. This phenomenon is expected to reduce soil water consumption, and fallen leaves could reduce surface soil water evaporation.

Gradients in the isotopic composition of water in soil profiles arise owing to differences in the seasonal input of rainwater into the soil and evaporation in the surface layers^28^. Due to the low rainfall in the dry season, evaporation is the key factor affecting the soil water isotope composition. The enrichment of soil water for δD and δ^18^O (especially in the surface soil layer) was greater in Rm than in HFAs, indicating that Rm soil experienced more evaporation. Since the gain in undergrowth coverage is predicted to decrease water evaporation relative to that of bare land^29^ and to capture more rain and fog water via interception^26^, *F. macrophylla* planted between the double hedgerows in rubber plantations could promote more soil water maintenance in HFAs than Rm. Increased soil water can ensure that plants uptake sufficient water, especially in the dry season, and is conducive to the migration and diffusion of nutrients^30^.

Water potential is the key physiological parameter in plant–water relationships, and fluctuations in shoot water potential are determined by transpiration and hydraulic conductance if the soil water potential surrounding the roots remains constant^31^. *Ψ*_pd_ is often used as a reliable indicator of the average soil water potential surrounding roots, and *Ψ*_md_ corresponds to the maximum transpiration^32^. A previous study^33^ demonstrated that well-watered soil conditions for rubber trees correspond to predawn values between −0.3 and −0.4 MPa, so the conditions of the soil layer in which rubber trees occupied at both sites were well-watered in our study (Fig. 7e). There were no significant differences in *Ψ*_pd_ among rubber trees and *F. macrophylla* in HFAs since they were in the same site. However, the slight seasonal oscillation in *Ψ*_pd_ of rubber trees accompanied by an almost invariable *Ψ*_pd_ of *F. macrophylla* (Fig. 7e) implied competition for water among the species owing to overlapping roots. Drought generally decreases leaf water potential, and this has been observed in many studies^34^,^35^. In HFAs, the seasonal variation in *Ψ*_pd_ and *Ψ*_md_ of *F. macrophylla* was not significant during the investigation, indicating that it is an isohydric species that allowed plant suffered little drought stress by a tight control of transpiration through stomatal closure^36^. In contrast, seasonal variation in *Ψ*_md_ of rubber trees indicated that it was an anisohydric species which has less stringent control by stomata. So, that somewhat explains why defoliation is a strategy to prevent excessive dehydration in rubber trees because only leaf yellowing and shedding drastically reduces the total hydraulic conductance of leaves, which could contribute between 40% and 80% of the whole-plant hydraulic conductance^37^,^38^. Actually, rubber tree defoliation occurred from mid-November to late February^39^, but the magnitude (i.e., the rate and quality) was initially too low. The magnitude gradually increased with leaf yellowing/reddening and reached a maximum about two weeks before mid-February, when leaf flushing occurred. A previous study supports our inference that rubber trees are anisohydric^40^, but other studies do not^33^. The differences among studies are mainly related to weather conditions. We measured shoot *Ψ*_md_ in August 2013 and November 2014, on mainly rainy/cloudy days. In addition, rubber trees in Rm suffered more drought stress than those in HFAs in the dry season (e.g., little rainfall in January 2014). *F. macrophylla*, which was planted with rubber trees, had little negative effect on the water requirements of the rubber trees as evidenced by the lack of a difference in drought stress in HFAs and Rm, even under the species competition (Fig. 7e).

According to the results of the IsoSource model, *F. macrophylla* mainly relied on surface soil water (<30 cm depth), and rarely or partially took up deep soil water (i.e., 30−50 cm in depth, see Fig. 5) when drought stress occurred (e.g., in January 2014, see Fig. 1). Rubber trees in Rm also depended on surface soil water, unless the surface soil drought occurred in the dry season. Despite the prevalence of feeder roots of rubber trees in the top 30 cm of the soil, there is no reason to believe that those in deeper layers are less efficient absorbers than those nearer to the surface^27^. Previous studies have also found that rubber trees efficiently use the available water in the root zone^19^,^39^. Similar studies investigating other plant species in a seasonally dry area have also concluded that plants can uptake water within the deep soil layer during the dry season^25^,^41^,^42^. However, rubber trees in HFAs would also uptake deep soil water in rainy season (e.g., within 30−50 cm depth in November 2013, see Fig. 5). To absorb sufficient water, rubber trees have to avoid unnecessary competition for surface soil water, which was occupied by the interplanted species, and then absorb deep soil water to meet water demands. For plants, competition could lead to two main types of evolutionary responses: increasing competitive ability and minimizing competitive interactions^43^. Obviously, the latter is more consistent with rubber trees in our study.

Some studies have suggested that roots can detect and avoid neighboring roots, and thus segregate spatially in territories^44^. The ability to discriminate the roots of other plants could reduce wasteful allocation to competition and allow greater resource availability for other functions, including greater reproductive output. However, unlike animals, for which niche specialization is widely thought to be a common adaptation to minimize interspecific competition, the ability to minimize interspecific competition is relatively limited in plants because they depend on nearly identical resources, and niche specialization is less likely^45^. Accordingly, competition cannot be completely avoided. Therefore, rubber trees absorbed a sufficient quantity of water by changing the relative use of each water source, instead of by abandoning regions of competition. The expansion of the water absorption zone not only weakened interspecific competition, but also minimized intraspecific competition. This interpretation is supported by the results of previous studies^46^. The proportion of water uptake indicated significant, strong plasticity in water uptake by rubber trees to avoid adverse factors (not only seasonal drought, but also interspecific competition).

The natural abundance of plant leaf δ^13^C, which may be a useful indicator of long-term WUE, has been examined extensively; it typically refers to the ratio of water used in plant metabolism to water lost by the plant through transpiration^47^. In general, WUE is defined as the ratio of biomass produced to the rate of transpiration, or as the ratio of the rate of carbon assimilation (photosynthesis) to the rate of transpiration, and is cited as a response mechanism of plants to soil water deficits and drought tolerance. The main benefits of increased WUE of plants in agricultural systems are typically increased yield and decreased transpiration-induced water loss^48^. The availability of water was not only affected by drought, but also by below-ground interspecific competition since soil water in HFAs was much more abundant than in Rm. That is, interspecific competition among species would improve plant WUE. Some studies not only support this viewpoint, but have also suggested that low WUE confers a competitive advantage when water is abundant^49^,^50^. That explains why *F. macrophylla* could always occupy surface soil water; its WUE was lower than that of rubber trees, especially in the rainy season. It is unexpected that rubber trees showed weakness in the face of *F. macrophylla* given its well-developed root system and its function as a water pump. In addition, the WUE of rubber trees in HFAs was always higher than in Rm, indicating that rubber trees in HFAs had higher productive potential for a lower supply of water. Since rubber tree evapotranspiration is energy-limited during the rainy season^19^, which means the maximum transpiration rate would occur in this season, a high WUE implies higher carbon assimilation (photosynthesis) ability. During the dry season, water consumption is mostly governed by environmental variables, so a high WUE ensures normal plant growth during drought conditions. In other words, higher WUE of rubber trees in HFAs is associated with higher productivity and less water waste relative to Rm. Moreover, plants in HFAs showed strong WUE stability, despite an extreme weather event in November 2013, since environmental factors (e.g., moisture and temperature) affect δ^13^C greatly^47^. HFAs can provide and maintain a stable internal microclimatic environment for plants.

C and N are the most essential elements for plants. Plants obtain C from the air and N from soil, fertilizer, and manure. Usually, leaf C and N concentrations indicate carbon fixation and photosynthetic capacity^51^. C from the air enters leaves as carbon dioxide (CO_2_), which is highly dispersive in the atmosphere, providing equal opportunities for plants to access^27^. This may explain why there was no significant difference in the leaf C concentration of rubber tree among sites. N from fertilizers, which is point-applied on surface soil in March and August at a dose of approximately 0.1 kg N per tree hole per year in the study area^52^, is mainly absorbed by plants through mass flow^53^. Indubitably, water is the most important carrier of N nutrients in soil.

Fine/feeder roots are the main organ of plants that absorb water and N. Therefore, the soil depths at which plants uptake water actively are also the main areas at which plants absorb N^28^. The close proximity of roots at different depths increases nutrient absorption by reducing nutrient leaching^27^. Since there was more runoff water in the rainy season, much of the mineral content/nutrients would be leached out of the soil. However, undergrowth coverage, which can reduce runoff water, and the deeper water uptake layer in HFAs would reduce nutrient leaching and thus help rubber trees absorb more N relative to rubber trees in Rm. However, in the dry season, the shallower water uptake layer of rubber trees in Rm promote nutrient uptake owing to the low water mobility in surface soil. That explains why the leaf N concentration of rubber trees in HFAs is higher in the rainy season, but lower in the dry season relative to rubber trees in Rm. Legume cover crops can help build up a pool of nutrients in the topsoil^27^. So the leaf N concentration of rubber trees in HFAs had less seasonal variation than those in Rm.

In addition, plant growth and defense are both fueled by compounds synthesized from a common pool of carbon and nitrogen, implying competition for carbon and nitrogen allocation to metabolism, and the C:N ratio of plant organs is often regarded as a convenient indicator of growth and quality^54^ and nitrogen use efficiency^55^. Typically, a higher C:N ratio indicates a higher nitrogen use efficiency, but lower growth rate^56^. Plant growth occurs mainly via reproductive and vegetative growth. A relatively higher C:N ratio is expected to promote reproductive growth and to decrease vegetative growth, and vice versa^57^. Since the C:N ratio decreased from the rainy season to the dry season, that mean vegetative growth (i.e., root, stem, and leaf growth) gradually increased. In the dry season, root growth was obviously predominant since defoliation occurred and rubber tapping activity was minimized. The leaf N concentration of rubber trees increased as the blade quantity decreased, which could mean that rubber trees need to maintain/enhance leaf photosynthesis to provide sufficient energy and materials for root growth. The decrease in the leaf C concentration could somewhat explain the distribution of C to root growth to facilitate water and nutrient absorption. This trade-off can be explained by four main hypotheses: the carbon-nutrient balance, the optimal defense theory, the protein competition model, and the growth-differentiation balance^54^. Root growth of rubber trees is more vigorous in Rm than in HFAs in the dry season for drought resistance. However, the relatively low leaf C:N ratio of rubber trees in HFAs indicated that rubber trees had a higher growth rate and reduced reproductive growth via promotion of vegetative growth to ensure more latex production relative to that of Rm in the rainy season. A previous study verified that intercrop *F. macrophylla* can promote the growth of rubber trees based on root, stem, and leaf biomass as well as the diameter at breast height, crown breadth, and height estimated over three years^58^. Although rubber trees in HFAs had a similar seasonal physiological rhythm (except WUE) as those in Rm, the variation was smaller during the investigation, indicating strong tolerance to climatic or environmental factors.

Overall, rubber trees in Rm heavily relied on shallow soil water (<30 cm), and adjusted their water utilization strategy to use deep soil water (30 cm) when shallow soil was insufficient, e.g., in the dry season. This plasticity was also important in HFAs, where there was interspecific competition for water between *F. macrophylla* and rubber trees. To avoid intense competition with the interplanted species for water, rubber trees expanded their water absorption zone to the deep soil layer. This response not only satisfied the demand of water by rubber trees, but also avoided excessive intraspecific competition and expanded the nutrient absorption zone to facilitate nutrient uptake by rubber trees. In addition, there was no evidence that this inevitable competition had a negative effect on rubber trees. In contrast, the competition greatly improved the WUE of rubber trees to reduce excessive water use; this is beneficial for water conservation in the rainy season, and ensures a normal water demand for plant growth under water shortages in the dry season. The HFAs can additionally reduce soil moisture evaporation via undergrowth coverage and can thus greatly conserve soil water and help maintain local microclimatic stability, despite in face of the adverse factors. But rubber trees in Rm were sensitive to environmental factors, irrespective of extreme weather events and serious seasonal drought. In summary, the higher system stability, tolerance stability, and potential productivity of HFAs relative to Rm indicate great improvements in rubber plantations. These results suggest that rubber trees planted with *F. macrophylla* have more beneficial effects than single cropping.

## Methods

### Study site

The study site was located in the Xishuangbanna Tropical Botanical Garden (XTBG; 21°55′39″N, 101°15′55″E) in Yunnan, southwestern China. The annual mean temperature at the site is 22 °C and the mean rainfall is 1,496 mm. Three seasons defined in previous studies are apparent in this region, i.e., the rainy season (May–October), fog-cool season (November–February), and hot-dry season (March–April)^5^,^12^. In the rainy season, rainfall accounts for approximately 84% of the annual total and the mean temperature is the highest (25 °C). The fog-cool season is the coolest period, with a mean temperature of 17 °C; there is dense fog in the morning and night and almost no rainfall. The subsequent hot-dry season is a transitional period, with less rainfall and a higher average temperature (22 °C). The fog-cool and hot-dry seasons are collectively referred to as the dry season owing to the obvious lack of rainfall. During the late dry season, rubber trees suffer serious drought stress because the soil moisture under the rubber monoculture approaches the permanent wilting point^5^,^59^.

Observations were conducted in a typical catchment (19.3 ha) covered with a 25-year-old rubber monoculture (clone PB86) that was designed in a modified spatial arrangement (i.e., double rows planted at 2 × 4.5 m separated by 14-m-wide inter-rows). The catchment spanned an altitudinal range of approximately 600−650 m, and a slope which was about 27−31 degrees. Soil under the rubber monoculture was approximately 2 m deep. The parent material at a depth of 2 m consisted of a 30−40-cm-thick layer of gravel deposited by a distributary of the Mekong River. Two planting patterns were selected for the study, Rm and HFAs. The intercropping species (*F. macrophylla*) was planted in seven lines at a density of 0.7 m × 1.0 m for about six years in 14-m inter-rows. In order to reduce error, the sites we selected had a common north-facing slope. The distance between the two sites was about 300 m and the difference in altitude was negligible.

### Sampling and measuring methods

Water samples for the isotope analysis were collected from rainwater, plant xylem, and soil. Samples for large rain events were routinely collected at a weather station from June 2013 to May 2014. Rainwater samples were collected immediately from a rain gauge after rain ceased in the dry season. Rainwater samples were stored in 2-ml screw-cap plastic vials, wrapped in Parafilm and frozen until analysis.

Plant and soil samples for the isotope analysis were collected separately during the rainy season (August 24, 2013), the fog-cool season (November 23, 2013), the late fog-cool season (January 19, 2014), the hot-dry season (March 10, 2014), and the early rainy season (May 5, 2014). Each sampling date could obviously representative of the particular season. At midday on each sampling date, plant xylem samples were obtained from each of three randomly selected rubber trees and *F. macrophylla* trees at each sampling site. For each sample, xylem tissues were obtained immediately by extracting small cylinders of wood from the trunk with an increment borer for rubber trees, and by cutting suberized mature stem segments from each of the four cardinal directions for *F. macrophylla* when possible. All green stem tissues were removed to avoid contamination of xylem water^60^. Upon collection, the clipped stem samples were immediately placed in 10-ml screw-cap glass vials, sealed with Parafilm, and then frozen (−20 °C) in the laboratory until water extraction.

Soil samples were collected with a hand-operated bucket auger. At each sampling date, three locations per sampling site within the planting line between the selected trees were randomly chosen. Since the feeder roots of mature rubber trees are mostly found in the upper 30 cm of the soil and there was little variation in the concentration of feeder roots among the inter-rows in the mature plantation^27^,^39^, the soil samples were collected from the soil layers of 0–5, 5–15, 15–30, 30–50, 50–80, and 80–110 cm depths at each location and divided into two parts. One for measuring the gravimetric SWC, and the other one for determining the soil water isotopic composition. We divided the soil profile into six unequally-spaced layers because the change of soil water isotopic composition gradually decrease from surface layer to deep layer owing to that surface soil was easily affected by environmental factors. Soil samples were stored as previously described for stem samples. Xylem and soil water was extracted using a cryogenic vacuum distillation system^60^ at the Central Laboratory of XTBG.

Predawn and midday leaf water potentials (i.e., *Ψ*_pd_ and *Ψ*_md_) were measured using a Pump-up pressure chamber (Pump-Up, PMS, Albany, OR, USA). *In situ* measurements were performed after cutting 3 to 6 leaves taken from sunny positions on each sampled tree between 4:00 and 6:30 for *Ψ*_pd,_ and between 12:30 and 14:30 for *Ψ*_md_. Both *Ψ*_pd_ and *Ψ*_md_ were measured 3−5 times. Leaves were also collected to determine δ^13^C and the total C and N concentrations, and were then dried to a constant mass and homogenized to a fine powder under an 80-mesh sieve.

The δD and δ^18^O values of the water samples and the δ^13^C of leaf samples were measured by a stable isotope ratio mass spectrometer (IsoPrime100, Isoprime, Stockport, UK) at the Central Laboratory, XTBG, with accuracy less than 2‰ and 0.3‰ for hydrogen and oxygen isotopic analyses respectively. Each isotope ratio is expressed as a ‰ relative to V-SMOW for H and O, and expressed as a ‰ relative to v-PDB for C. The total C and N concentrations of leaf samples were measured using Vario MAX cube (Elementar, Hanau, GER).

## Calculations

The relative contribution of each water source was calculated using an IsoSource mixing model^23^. For *F. macrophylla*, only the soil layers above 80 cm depth were considered owing to its rhizosphere depth. We did not consider the utilization of rain water since rainfall was not the direct water source for the plants in our study and the rain events were not continuous. There exist isotopic mixture and fractionation in the process of rain infiltration which will affect the result if we consider recent rainfall as one water source directly. This model provides a range of possible solutions instead of a distinct solution. Hence, the average contribution of each source is presented with a range^24^. Combinations that summed to the observed mixture isotopic signatures were calculated within a small tolerance (i.e., 0.05%) and examined in small increments (i.e., 1%). Finally, we combined several water sources which show similar isotopic composition in water by a posteriori method^24^ suggested by Phillips *et al.* owing to simplify the mixing analyses of the overabundance of sources.

There is a strong relationship between ∆^13^C and WUE (i.e., the molar ration of photosynthesis (A) to transpiration (E), or A/E)^25^,^47^,^60^, especially for plants with C_3_ photosynthesis. Since the carbon incorporated into leaf tissues is assimilated over a considerable period of time and under more than one set of environmental conditions, measurements of ∆^13^C provide an average estimate of C_i_/C_a_ (C_i_ and C_a_ refer to the ambient CO_2_ concentrations in the internal gas space of the leaf and the surrounding atmosphere, respectively) and, therefore, an index for time-integrated (and flux-weighted) plant metabolism. Because both ∆^13^C and A/E are functions of C_i_/C_a_, ∆^13^C can be used to estimate time-integrated or flux-weighted long-term A/E for the same foliage. As such, ∆^13^C should be considered an absolute index of integrated C_i_/C_a_, but a relative index of A/E^32^. Additionally, ∆^13^C is inversely proportional to A/E^32^. Accordingly, high WUE in leaves inevitably leads to low ∆^13^C values. This is the opposite of δ^13^C-based measurements, for which favorable WUE estimates are associated with high δ^13^C (less negative) values. Since the δ^13^C of the atmospheric CO_2_ is nearly constant in the global troposphere within a given year and Farquhar provide an estimate atmospheric composition of −8% for those field-grown plants^47^ where the gradients of atmospheric CO_2_ concentrations under the canopy are found to be smaller, we can compare the WUE of plants in our study by comparing their leaf δ^13^C.

### Statistical analyses

Differences in the isotopic composition of the xylem sap, leaf δ^13^C, leaf C and N concentrations, and shoot water potential of rubber trees between seasons and sites were evaluated using general linear models (GLM) with season and site as fixed effects. Differences between rubber trees and the interplant were analyzed by GLM models with season and species as fixed effects. Seasonal differences in the isotopic composition of soil water between sites and depths were analyzed by GLM models with season, site, and depth as fixed effects. The same analysis was used for SWC. To analyze differences in the isotopic composition of the xylem sap, leaf δ^13^C, C and N concentrations, C:N ratio, and shoot water potential, GLM models were run with season as a fixed effect separately for each site and species. If the result was significant, differences between groups were evaluated using post-hoc honestly significant difference tests (i.e., Tukey tests and Duncan’s new multiple range tests). If the interaction effect was significant, a simple effects analysis was performed. All statistical analyses were implemented in SPSS 21.0 (SPSS Inc., Chicago, IL, USA).

## Additional Information

**How to cite this article**: Wu, J. *et al.* Below-ground interspecific competition for water in a rubber agroforestry system may enhance water utilization in plants. *Sci. Rep.* **6**, 19502; doi: 10.1038/srep19502 (2016).

## Figures and Tables

**Table 1:** *F-*values estimated using GLM models for the differences in δ^13^C value, C and N concentration, C:N ratio, and shoot water potential.

	Tested effects	*d.f*	δ^13^C	C	N	C:N	*Ψ*_pd_	*Ψ*_md_
(A)	Season	4	13.31**	6.53**	31.98**	37.22**	13.02**	65.15**
	Site	1	62.03**	0.06	0	1.77	0.69	1.81
	Season × Site	4	2.47	0.38	9.52**	10.3**	0.57	30.88**
(B)	Season	4	19.79**	9.67**	6.35**	13.34**	3.96*	21.2**
	Species	1	275.88**	5.49*	108.93**	158.19**	2.08	0.53
	Season × Species	4	2.14	0.53	2.2	5.02**	1.35	11.9**
(C)	Season	4	8.83**	4.81*	25.54**	25.04**	10.45**	107.96**
(D)	Season	4	7.23**	2.8	10.01**	19.41**	5.16*	34.71**
(E)	Season	4	20.09**	17.26**	2.1	3.16	2.2	1.3

The results include the following: (**A**) the effect on *H. brasiliensis* at both sites; (**B**) the effect on all plant in HFAs; (**C**) the effect on *H. brasiliensis* in Rm; (**D**) the effect on *H. brasiliensis* in HFAs; (**E**) the effect on *F. macrophylla* in HFAs. Rm and HFAs indicate rubber monoculture and *Hevea-Flemingia* agroforestry system.

^*^P < 0.05, ^**^P < 0.01.
